# Physiological benefits of protected AD3E on nutrient digestibility, hematology, and small intestinal morphology and carcass characteristics in fattening lambs

**DOI:** 10.1016/j.vas.2026.100756

**Published:** 2026-07-01

**Authors:** Danyal Zarrin Kelk, Mohammad Ebrahim Nooriyan Soroor, Fardin Hozhabri, Hadi Cheraghi, Khoda Bakhsh Rashidi

**Affiliations:** a Department of Animal Sciences, Faculty of Agricultural Sciences and Engineering, Razi University, Kermanshah, Iran; b Department of Animal Sciences, Faculty of Agriculture, Bu-Ali Sina University, Hamadan, Iran; c Department of Clinical Sciences, Faculty of Veterinary Medicine, Razi University, Kermanshah, Iran; d Adipose Tissue and Oils Research Center, Health Technology Research Institute, Kermanshah University of Medical Sciences, Kermanshah, Iran

**Keywords:** Lambs, Intestinal histomorphometry, Protozoa population, Rumen fermentation, Vitamin, Weight gain

## Abstract

Adequate intake of fat-soluble vitamins, including vitamin A, D_3_, and E, is essential for fattening lambs due to their roles in immune competence, metabolic regulation, and antioxidant protection. Conventional diets often provide inconsistent levels of these vitamins, making supplementation necessary. The present study was conducted to investigate the effect of different levels of rumen-protected AD_3_E supplementation on performance, rumen fermentation, feed digestibility, quantitative carcass characteristics, and small intestinal morphology of fattening lambs. Twenty-four male lambs (4-month-old) with a body weight of 34.11 ± 2.2 kg were divided into three groups in a completely randomized design for 70 days. The experimental treatments included a control group fed a basal diet (without vitamin supplementation), a second group fed a basal diet plus 1 kg/t of vitamin AD_3_E, and a third group fed a basal diet plus 2 kg/t of vitamin AD_3_E. Growth performance, nutrient digestibility, rumen fermentation parameters, and carcass traits were evaluated. The results showed that the use of protected AD3E improved the daily weight gain and feed conversion ratio of male lambs in the two vitamin-receiving groups compared to the control group (P < 0.05).The final weight of lambs receiving the protected vitamin supplement was higher than that of the control group (P < 0.05). Digestibility of nutrients remained unaffected. In comparison with the control group, rumen protozoa (total population and Entodinium) significantly increased in lambs that received vitamin at a level of 1 kg per ton (P < 0.05). Blood parameters showed alterations, including reduced glucose at 1 kg/ton, increased total protein and reduced cholesterol at 2 kg/ton, and elevated hematocrit and hemoglobin with supplementation. Small intestinal morphology showed no significant changes in villus height or crypt depth. Carcass traits were unaffected. In general, the results showed that the use of protected vitamin AD_3_E supplement in lamb diets improved the performance of fattening lambs (Final Body Weight (kg), Total Weight Gain (kg) and Daily Weight Gain (g)) through a positive effect on rumen fermentation, although it did not affect diet digestibility.

## Highlights

• Protected AD_3_E boosted daily gain by 16.5% via rumen effects.
• Improved Feed Conversion Ratio (FCR) by 12% at 1 kg/ton without altering intake.
• Enhanced rumen ammoniacal nitrogen (NH_3—_N) and protozoa by up to 35%.
• Novel protected form advances lamb nutrition efficiency.
• Lower glucose at 1 kg/ton; Hemoglobin / Hematocrit (Hb/Hct) overall.

## 1 Introduction

Fattening lambs requires vitamins for optimal performance and health, with each vitamin serving a unique, irreplaceable function (Paulo et al., 2022). Due to wide variations in vitamins A, D_3_, and E content in typical livestock feeds, especially harvested forages, and factors affecting their utilization and bioavailability, animals cannot synthesize these fat-soluble vitamins (NRC., 2001). The vitamins are prone to rumen microbial degradation, reducing bioavailability; thus, protected forms (e.g., encapsulated to bypass rumen) ensure post-ruminal absorption and efficacy (Sarkar et al., 2026). This degradation necessitates protected supplementation to meet physiological needs and sustain high production (Sarkar et al., 2026), as water-soluble vitamins and vitamin K are sufficiently synthesized by rumen microbes (Krogstad et al., 2025). Per NRC recommendations, fattening lambs need 3000 IU retinol, 300 IU tocopherol, and 555 IU cholecalciferol per 100 kg body weight daily (NRC, 2007). Vitamin E, essential for sheep but not stored in tissues, is particularly critical for young lambs at 10–60 mg per kg dry matter (NRC, 1985). These vitamins collectively support animal health (McDonald et al., 2010).

Standard feeds for fattening lambs often lack sufficient A, D_3_, and E, limiting alfalfa use due to high costs; thus, dietary addition can enhance health and growth (McDonald et al., 2010). Vitamin A maintains epithelial cells, organ function, and growth (Song et al., 2023; Tanumihardjo, 2011). Vitamin D_3_ aids bone formation, calcification, and absorption of calcium, phosphorus, iron, zinc, and magnesium (Al-Asadi et al., 2020). Vitamin E, a natural antioxidant, promotes growth, reproduction, and immune response, and reduces oxidation of cell wall phospholipids in dairy cattle (Xiao et al., 2021) and livestock (Shastak et al., 2023) .

Studies on beef steers (Gorocica-Buenfil et al., 2007; Knutson et al., 2020) and on calves (Scapol et al., 2023) show vitamin A supplementation has no significant effect on daily weight gain, dry matter intake, or FCR, even at low levels. Vitamins influence carbohydrate and fat metabolism, potentially increasing feed intake via reduced blood glucose and enhanced appetite, while deficiencies may reduce consumption (McDowell et al., 2008). Vitamins B, C, and D boost dry matter decomposition, rumen metabolism, and fermentation (Witariadi et al., 2022). Vitamin E elevates total volatile fatty acids, acetate production, acetate-to-propionate ratio, and reduces butyrate in goat rumen fluid in vitro (Hou et al., 2013). In dairy cows, AD_3_E improves immune and antioxidant systems, enhancing GPx and SOD, without affecting carcass traits (Wang et al., 2022a). The use of vitamin D_3_ (7.5 × 10^6^ IU) (Baldin et al., 2013) and 2 × 10^6^ IU (Lobo-jr et al., 2012) has shown no effect on growth performance, carcass characteristics, and meat quality in steer . The studies by Ramos et al. (2024) on four groups of fattening calves (control, vitamin AD_3_E, vitamin B, and AD_3_E+B; ordinary type) have shown that supplementation with conventional vitamin AD_3_E in the diet of fattening calves, compared to the other groups, had no effect on rumen pH but did increase intramuscular fat (marbling).

The supplementation levels were designed to provide protected forms exceeding basal NRC (2007) requirements by approximately 2–4-fold to compensate for potential rumen losses, without inducing hypervitaminosis, as supported by recent studies on rumen-protected vitamins in ruminants (Shastak et al., 2023). Because vitamins are highly sensitive to rumen degradation, they are coated (Santschi et al., 2005). Despite known roles of vitamins A, D_3_, and E, gaps exist in protected forms' effects on fattening lambs' rumen-protected delivery to enhance bioavailability amid high-concentrate diets. This study addresses this by evaluating 1 and 2 kg/ton protected AD_3_E on growth, digestibility, rumen fermentation, blood profile, intestinal morphology, and carcass traits. Protected AD_3_E is vital to overcome rumen degradation, improving nutrient efficiency and microbial balance in high-concentrate diets, with potential applications in lamb nutrition. This study's strengths include its comprehensive assessment of physiological parameters in Mehraban lambs, filling knowledge gaps on protected forms' impacts for practical ruminant feeding strategies. Thus, we hypothesized that the supplementation of fat-soluble vitamins (AD3E), will improved lamb performance, rumen fermantaion and carcass characteristics.Since little is known about protected AD_3_E's (Rumen Protected with Fat Coating)effects on fattening lambs, this research justifies investigating dose-dependent benefits on performance, digestibility, fermentation, and carcass traits.

## 2 Materials and methods

These experiments were conducted in accordance with established animal welfare guidelines, and all experimental protocols adhered to and were approved by the Ethics Committee of Razi University, Kermanshah, Iran (ethics approval number: IR.RAZI.REC.1402.052). After conducting the experiments and submitting a full report with details about the experimental methods to the relevant committee, the committee confirmed the certificate number IR.RAZI.REC.1402.063 that the study complies with all regulations, in your manuscript.

### 2.1 Animals and treatments

A total of 24 four-month-old male Mehraban lambs with an initial average weight of 34.11 ± 2.2 kg (SD low, CV <7% to minimize variability), were housed individually for 70 days in a completely randomized design in 3 groups with 8 replications. During the adaptation period (14 days), all health operations such as vaccination, control of internal and external parasites, and adaptation to feed were performed. Based on NRC (2007) and to increase daily weight by 300 g, the sheep's diets were adjusted with a composition of 30% forage and 70% concentrate, including 14% protein (Table 1). The daily ration consumed by each animal was at the level of appetite, and the daily food required by the sheep was distributed at 8:00, 14:00, and 19:00. Fresh water was constantly available to each lamb throughout the entire period. Experimental treatments included treatment one (control): basal diet, without vitamin supplementation; Treatment two: basal diet + vitamin AD_3_E mixed in the feed at one kg/ton; and treatment three: basal diet + vitamin AD_3_E mixed in the feed at two kg/ton. The protected AD_3_E (Rumen Protected with Fat Coating) supplement included 3.500.000 IU/kg, 1.200.000 IU/kg, 25 IU/kg Vitamin A, D_3_, and E, respectively, and 300 mg selenium/kg (Behdam Roshd Khorasan, Iran). In the previous study (Nazari Darabkhani et al., 2024), the recommended consumption doses based on NRC had no effect on the growth of fattening lambs. On the other hand, the exact requirement of fattening lambs for vitamin AD3E is not known; therefore, these amounts have been selected.The methods described in AOAC International were used to determine the DM crude protein and ash (Agroindustriais, 2013). The neutral detergent fiber (NDF) and acid detergent fiber (ADF) were determined using the protocol of Van Soest et al. (1991). Metabolizable energy calculated based on the diet ingredients and NRC (2007) tables data (Table 1).

**Table 1:** Components and chemical compositions of the diets used in the experiment.

Diet components (%)	Treatments		
	1	2	3
Alfalfa (%)	20	20	20
Wheat Straw (%)	10	10	10
Barley (%)	34	34	34
Corn (%)	20	20	20
Soybean meal (%)	13.50	13.50	13.50
Salt (%)	0.3	0.3	0.3
Dicalcium phosphate (%)	0.3	0.3	0.3
Buffer (%)	1	1	1
Mineral supplement (%)	0.9	0.8	0.7
Vitamin supplement Chemical composition (%)	0	0.1	0.2
Dry matter	88.81	88.81	88.81
Metabolizable energy (Mcal/Kg)	2.64	2.64	2.64
NEm (Mcal/Kg)	1.795	1.795	1.795
NEg (Mcal/Kg)	1.074	1.074	1.074
Crude protein (%)	14.28	14.28	14.28
Crude Fiber (%)	18.38	18.38	18.38
Ash (%)	7.56	7.56	7.56
NDF (%)	32.04	32.04	32.04
ADF (%)	24.58	24.58	24.58
Additive vitamin (IU/kg of diet)			
A (IU/kg of diet)	0	478091	937840
D3 (IU/kg of diet)	0	163917	321545
E (IU/kg of diet)	0	3.415	6.69

Treatments 1, 2, and 3, respectively, represent: control without supplementation, diet containing one kilogram per ton of protected AD_3_E supplement, and diet containing two kilograms per ton of protected AD_3_E supplement. Each kilogram of mineral supplement contains: 96 g of phosphorus, 196 g of calcium, 71 g of sodium, 19 g of magnesium, 3 g of iron, 0.3 g of copper, 2 g of manganese, 3 g of zinc, 0.1 g of cobalt, 0.1 g of iodine, 0.001 g of selenium, and 0.4 antioxidants. Vitamin supplement AD_3_E contains 3.500,000 IU of vitamin A, 1.200,000 IU of vitamin D_3_, 25 IU of vitamin E, and 300 mg of selenium.

### 2.2 Performance

Daily feed was adjusted according to the recommendation of NRC (2007) and placed in the animal's manger so that 5% of the feed remained as residue in the manger of each animal (n = 24). Every morning before feeding the morning meal, the feed residue of each lamb was collected from the manger, and then fresh feed was replaced. To calculate the feed consumption, the amount of feed given and the daily feed residue of each lamb were recorded throughout the entire experimental period. The dry matter intake (DMI) of each lamb was determined based on the subtraction of the daily feed residue of each animal from the daily dry matter offered. To estimate the performance parameters of each lamb, the animals were weighed weekly after 8 h of starvation and before feeding the morning meal. During the entire experimental period, the total weight gain of each lamb was obtained by subtracting the final weight from the initial weight. The daily weight gain of each lamb was also determined by dividing the total weight gain by the number of days of rearing (70 days). Feed conversion ratio (FCR) was obtained by dividing the total DMI of each lamb (Kg) by the total weight gain (Kg) during the fattening period. Also, the shrunk body weight, expected DMI, DMI Obs/DMI Exp, and NEm, NEg expected were calculated according to Urías-Estrada et al. (2023) (Table 2).

**Table 2:** Effect of varying levels of protected vitamin AD_3_E supplementation on the performance of fattening lambs.

Performance Parameters	Treatments*				*P-value*
	1	2	3	SEM	
Initial Weight (kg)	34.175	34.175	34.000	0.697	0.994
Initial SBW (kg)	33.41	32.30	33.34	0.683	0.777
Final Body Weight (kg)	51.950^b^	54.350^a^	54.950^a^	1.249	0.001
Total Weight Gain (kg)	17.77^b^	20.18^a^	20.95^a^	0.879	0.020
Daily Weight Gain (g)	273.4^b^	307.8^a^	318.8^a^	12.670	0.023
Feed Efficiency (gain-to-feed ratio)	0.135^b^	0.157^ab^	0.167^a^	0.006	0.050
Observed					
NEm (Mcal/Kg)	1.795	1.795	1.795	0.000	1.00
NEg (Mcal/Kg)	1.074	1.074	1.074	0.000	1.00
DMI (kg)	2.00	1.95	1.92	0.039	0.671
Expected					
NEm (Mcal/Kg)	2.038	2.198	2.246	0.078	0.550
NEg (Mcal/Kg)	1.251	1.387	1.429	0.062	0.503
DMI (kg)	1.592	1.718	1.756	0.623	0.550
Observed to Expected					
NEm	0.961	0.838	0.804	0.047	0.374
NEg	1.013	0.815	0.759	0.073	0.343
DM intake	1.256	1.135	1.093	0.0623	0.550

^a,b^ Different letters in each row indicate significant differences at the (*P* < 0.05) level.

Treatments 1, 2, and 3 represent, respectively: control without supplementation, diet containing 1 kg per ton of protected AD_3_E supplementation, and diet containing 2 kg per ton of protected AD_3_E supplementation.

Shrunk body weight (SBW).

Final body weight, the lamb was weighed following an 18 h fasting at the end of the experimental period.

### 2.3 Nutrient digestibility

At the end of the experimental period, five lambs (BW, 53.75 ± 1.43) were selected from each treatment and placed in metabolic cages for seven days (Table 3). Feed distribution and feces collection were performed every day at a specific time. The amount of feed consumed was calculated based on the amount of feed given and the amount remaining the next day. The amount of feed offered to the animals in the digestibility trial was based on the maintenance requirements of the animals. The feces of each animal were collected for 7 days. Approximately 100 g sample of feces from each animal was taken each day to determine dry matter, and a sample based on ten percent of daily feces was taken for subsequent experiments and stored at −20 °C. Samples were taken from the consumed feed and daily feed waste to determine the chemical composition (Agroindustriais, 2013). Fecal samples from each animal were thawed, homogenized, and subsampled for analysis. Total waste samples were similarly pooled, and a representative aliquot was retained.

**Table 3:** Effect of varying levels of protected vitamin AD_3_E supplementation on the feed digestibility.

Digestibility parameter	Treatments*				*P-value*
	1	2	3	SEM	
Dry matter (%)	88.52	89.83	85.50	8.3	0.98
Crude protein (%)	71.34^a^	74.60^a^	79.36^b^	0.86	0.01
Fat (%)	65.58	65.95	65.20	0.15	0.69
NDF (%)	69.14	66.42	67.86	3.04	0.69
ADF (%)	79.52	71.28	71.63	1.95	0.68

^a,b^Different letters in each row indicate significant differences at the (*P* < 0.05) level.

*Treatments 1, 2, and 3 represent, respectively: control without supplementation, diet containing 1 kg per ton of protected AD_3_E supplementation, and diet containing 2 kg per ton of protected AD_3_E supplementation.

### 2.4 Blood sampling and measurements

At end of experimental period, 08:00 am, blood samples were taken from jugular vein of all 8 lambs/treatment (n = 24) in heparinized (plasma) and non-heparinized (serum) tubes; then were centrifuged (3000 rpm, 10 min), and serum and plasma were stored at −20 °C until analysis including glucose, total protein, albumin, creatinine, triglyceride, cholesterol, HDL, LDL, ALP, ALT, AST, Hct, Hb, RBC, MCV, MCHC, and Plt, via an fully automatic cell counter (Boule Medical, Exigo Vet, Sweden) and auto-analyzer (Alpha-classic, Iran), and commercial kits (Pars Azmoon®, Tehran, Iran).

### 2.5 Fermentation parameters

The rumen fluid of each lamb was collected by esophageal tube three hours after the first meal (12:30 PM) and at the end of the period. To accustom the lambs to the process of collecting rumen fluid and removing saliva from the collected fluid, rumen fluid was collected from the lambs every day for three days before, and on the fourth day, the final rumen fluid sample was collected for the experiment. The pH of the rumen fluid was recorded by a pH meter (Hanna-USA Hl98103), ammonia nitrogen was measured according to Broderick and Kang (1980) using a spectrophotometer (Scan UV Visible, CARY100, VARIAN) at a wavelength of 630 nm and volatile fatty acids was measured according to Ottenstein and Bartley (1971) using GC (Unicam 4410, UK); 4-Methylvaleric acid (Sigma) was used as an internal standard.

The amount of energy loss in the form of methane gas produced was estimated based on the following relationships (Klasing, 2007).

(1) $$CH_{4\left(\text{MJ}/d\right)}=3.96\left(\pm 1.18\right)+0.561\left(\pm 0.130\right)\times \text{DM}I_{\left(\text{kg}/d\right)}$$

(2) $$CH_{4\left(\text{MJ}/d\right)}=2.70\left(\pm 1.38\right)+1.16\left(\pm 0.271\right)\times \text{DM}I_{\left(\text{kg}/d\right)}--15.8\left(\pm 6.86\right)\times EE_{(\text{kg}/d)}$$

Eq. (1) is based on a constant coefficient and DMI, and in Eq. (2), a constant coefficient, DMI, and crude fat of the feed, and the amount of methane gas produced was estimated in megajoules per day.

### 2.6 Protozoa enumeration

Identification and enumeration of protozoa were performed using Dinocapture software installed on a computer, a light microscope, and a hemocytometer slide according to the recommended method (Dehority, 2003). The hemocytometer slide was washed with distilled water and then dried with a soft paper towel. Then, the contents of a 50-mL Falcon tube were gently mixed. Using a syringe, a drop of the sample was transferred into the central cavity of the hemocytometer slide, the slide was placed on the counting slide, and counting was performed at a magnification of 10 times using the microscope. The genus of protozoa was calculated based on the number of ciliated areas and the distribution of cilia on the cell and their total concentration per mL of rumen contents.

### 2.7 Carcass characteristics, sampling, and morphometry of the small intestine

Three lambs were randomly (random selection ensured, avoiding bias.) selected per treatment independent of digestibility subset (n = 8), weighted and slaughtered at the end of the experiment in order to study the carcass characteristics and organ weights including post-slaughter weight, carcass weight, weight of wool and skin, carcass length, carcass width, thigh length, and hand length according to Asadian et al. (1996); besides, the small intestine of the lambs were then removed through dissection (Table 8). Then, a sample of approximately 5 cm from the duodenum, jejunum, and ileum was taken. The tissues were fixed in 10% formalin for four days, stained, and embedded in paraffin according to standard procedures (Yoon et al., 2013). A light microscope was used to take the pictures. Image software was used to measure papilla length, papilla width, and cript depth in the duodenum, jejunum, and ileum (Axiovision, 8.4; Carl Zeiss, Oberkochen, Germany).

### 2.8 Statistical analysis

This experiment was conducted in a completely randomized design with three treatments and eight replications (lamb) per treatment. The data obtained for each trait were analyzed using SPSS software (version 27) at the level (α = 0.05) and the following statistical model. Duncan's multiple range test was used for post-hoc comparisons following ANOVA, as it is suitable for equal group sizes and controls type I error effectively (Montgomery, 2017). Data normality and homogeneity were confirmed via Shapiro-Wilk (P > 0.05) and Levene's tests (P > 0.05); no transformations were needed. The statistical model was as follows:

$$y_{\text{ij}}=\mu +T_{i}+e_{\text{ij}}$$

where: y_ij_ = i^th^ observation from j^th^ treatment, µ = total mean, T_i_ = i^th^ treatment and e_ij_ = residual effects.

## 3 Results and discussion

### 3.1 Performance

Table 2 demonstrates that the administration of protected vitamin AD_3_E significantly influenced various performance metrics in fattening lambs throughout the experimental period. The initial weights were comparable across all treatments, confirming uniformity in baseline conditions. However, the final body weights (FBW) were notably higher in treatments 2 and 3 compared to the control, implying that the vitamin supplementation facilitated greater growth. Total weight gain followed a corresponding pattern, with treatment 2 recording the highest gain, which was significantly greater than that of the control, while treatment 3 exhibited an intermediate response. Furthermore, average daily gain (ADG) was significantly improved in treatment 2 relative to the control, with treatment 3 again presenting intermediate outcomes. Therefore, dietary inclusion of the AD_3_E at 1 kg/ton led to significant increases in FBW and ADG (P < 0.05), with no detectable effects on DMI. The 16.5% improvement in daily gain observed at the moderate dose of vitamin underscores enhanced feed efficiency. A 12% improvement in feed conversion ratio (FCR) was observed with the inclusion of 1 kg/ton of AD_3_E (P = 0.03), indicating more efficient nutrient utilization. This finding supports the established role of vitamins in enhancing metabolic efficiency without increasing feed intake (McDowell, 2008). Therefore, vitamins may play an important role in the metabolism of carbohydrates, fats, proteins, and also the growth of the animal (McDowell et al., 2008)

**Table:** 

Performance parameters	Treatments			SEM	*P-value*
	1	2	3		
Initial Weight (kg)	34.17	34.17	34.00	0.69	0.99
Initial SBW (kg)	33.41	32.30	33.34	0.68	0.77
Final Body Weight (kg)	51.95^b^	54.35^a^	54.95^a^	1.24	0.01
Total Weight Gain (kg)	17.77^b^	20.18^a^	20.95^a^	0.87	0.02
Daily Weight Gain (g)	273.40^b^	307.80^a^	318.80^a^	12.67	0.02
Feed Efficiency (gain-to-feed ratio)	0.13^c^	0.15^ab^	0.16^a^	0.00	0.05
Observed					
NEm (Mcal/Kg)	1.79	1.79	1.79	0.00	1.00
NEg (Mcal/Kg)	1.07	1.07	1.07	0.00	1.00
DMI (kg)	2.00	1.95	1.92	0.03	0.67
Expected					
NEm (Mcal/Kg)	2.03	2.19	2.24	0.07	0.55
NEg (Mcal/Kg)	1.25	1.38	1.42	0.06	0.50
DMI (Kg)	1.59	1.71	1.75	0.62	0.55
Observed to Expected					
NEm (Mcal/Kg)	0.96	0.83	0.80	0.04	0.37
NEg (Mcal/Kg)	1.01	0.81	0.75	0.07	0.34
DM intake (kg)	1.25	1.13	1.09	0.06	0.55

^a,b^Different letters in each row indicate significant differences at the (*P* < 0.05).

Treatments 1, 2, and 3 represent, respectively: control without supplementation, diet containing 1 kg per ton of protected AD_3_E supplement, and diet containing 2 kg per ton of protected AD_3_E supplement.

Shrunk body weight (SBW).

Final body weight, the lamb was weighed following an 18 h fasting at the end of the experimental period.

Limited studies have investigated AD_3_E supplementation on animal fattening performance, and most studies have examined vitamins individually and reported conflicting results. Vitamin AD_3_E improved calf weight gain compared to the control treatment, and it was stated that this improvement in the group consuming vitamin AD_3_E could be related to the effect of vitamins A, D_3_, and E on increasing appetite and palatability of the diet, which increased dry matter intake and ultimately improved growth and performance of the animals under test (Habeeb et al., 2018). These researchers also stated that the significant improvement in calf weight gain due to vitamin A and E supplementation may be because these vitamins are involved in the synthesis of important coenzymes (NAD and FAD) that are responsible for biological oxidative processes that produce ATP required for the biosynthesis of proteins, fats, and carbohydrates. Similar to the findings of the present study, Al_Galiby & Al-khafaji also demonstrated in three groups of growing goat kids that intramuscular administration of vitamins AD_3_E twice a week significantly improved daily weight gain and total weight gain of growing goat kids (Al_Galiby and Al-khafaji, 2022). Vitamin E supplementation in Karya lambs showed no significant effect on daily gain but improved FCR by about 10%, suggesting vitamin E may enhance metabolic efficiency in fattening ruminants (Atay et al., 2009).

The improved growth metrics and FCR observed with protected AD_3_E supplementation can be attributed to three distinct yet synergistic physiological pathways: (1) endocrine regulation of the somatotropic axis (vitamin A), (2) enhanced skeletal mineralization and nutrient utilization (vitamin D_3_), and (3) cytoprotective antioxidant activity with secondary immunometabolic effects (vitamin E). The most clearly elucidated mechanism involves vitamin A (retinol) acting as a direct nutritional regulator of the somatotropic axis. Oka et al. (2004) demonstrated that animals receiving adequate vitamin A supplementation exhibited significantly higher serum IGF-1 concentrations and superior ADG compared to vitamin A-restricted counterparts, despite equivalent DMI and no detectable difference in growth hormone (GH); however, retinoic acid, a form of vitamin A, has also been shown to regulate growth hormone gene expression (Cheung et al., 2020). This indicates that vitamin A exerts its growth-promoting effect not by increasing GH secretion, but by enhancing hepatic IGF-1 synthesis and/or secretion. These findings are consistent with earlier molecular work in avian models demonstrating that vitamin A deficiency reduces IGF-1 gene expression in hepatic tissue (Fu et al., 2001). Given that IGF-1 is the principal mediator of GH-dependent somatic growth and nitrogen retention, the elevation of circulating IGF-1 provides a direct endocrine mechanism for the improved weight gain and feed efficiency observed in our study. Vitamin D_3_ (cholecalciferol) contributes to growth performance through its classical function in calcium and phosphorus homeostasis. The AD_3_E supplementation literature confirms that vitamin D_3_ increases the efficiency of intestinal calcium and phosphorus absorption, which is requisite for bone formation and calcification (Nazari Darabkhani et al., 2024). In rapidly growing fattening lambs, adequate skeletal mineralization is a permissive factor for lean tissue accretion. Beyond mineral metabolism, emerging evidence in ruminant nutrition indicates that vitamin D status influences gene expression related to muscle development and carcass composition (Hernández & Tamassia, 2025). Thus, improved FCR may reflect a reduction in the metabolic cost of skeletal maintenance, allowing a greater proportion of dietary energy to be partitioned toward muscle protein synthesis. Vitamin E preserves membrane fluidity and function by neutralizing reactive oxygen species generated during high metabolic flux. This cytoprotective effect is particularly relevant in fast-growing lambs, where oxidative stress can impair myocyte and hepatocyte function (Shojadoost et al., 2021). Furthermore, vitamin E exerts immunomodulatory effects that indirectly support growth. By reducing the expression of pro-inflammatory cytokines (e.g., IL-1β, IL-6) and enhancing T-lymphocyte (CD4⁺, CD8⁺) and B-lymphocyte responses, vitamin E mitigates the nutrient diversion associated with subclinical immune activation (Shojadoost et al., 2021). The reduction in serum BUN and altered ammonia nitrogen observed in rumen-protected AD_3_E treatments (Nazari Darabkhani et al., 2024) may reflect improved nitrogen retention and more efficient ruminal nitrogen partitioning, consistent with reduced catabolism of amino acids. Although these specific parameters were not directly reported in our present study, they are physiologically congruent with the observed 12–16.5% improvements in gain and FCR. The improvement in FCR without concomitant increases in DMI strongly indicates that the observed performance gains are driven by enhanced metabolic efficiency rather than increased intake. The combination of endocrine stimulation (IGF-1-mediated anabolism), improved skeletal mineral availability, and reduced oxidative/immune maintenance costs provides a coherent physiological explanation for why protected AD_3_E supplementation enables lambs to achieve greater tissue accretion per unit of feed consumed.

### 3.2 Digestibility

Table 3 indicates no significant effects of protected vitamin AD_3_E supplementation on nutrient digestibility, except for protein digestibility. Similarly, Kannan et al. (2024) showed that supplementation of 25-hydroxyvitamin D_3_ did not affect the digestibility of nutrients in goats. The dose-dependent increase in crude protein digestibility observed with AD_3_E supplementation at 2 kg/ton, but not at 1 kg/ton, can be attributed to several interconnected physiological and metabolic mechanisms, although direct evidence for protein digestibility responses specifically remains limited in the literature.

Vitamin A is essential for maintaining epithelial tissue integrity throughout the digestive tract, and Vitamin A and vitamin D regulate the expression of tight junction proteins on intestinal epithelial cells that are critical for barrier function in the gut (Cantorna et al., 2019). Vitamin A has an essential role in vision and cellular differentiation, the latter providing a unique core mechanism helping to explain the influence of vitamin A on epithelial barriers and alterations in the epithelial lining of vital organs occur early in deficiency, suggesting a potentially important role for the barrier function (McCullough et al., 1999). All-trans retinoic acid (ATRA) is crucial for maintaining the integrity of the gut epithelial barrier by regulating the expression of tight junction proteins such as zona occludens-1 (ZO-1), claudin-1, and occludin (Skawratananond et al., 2025). Adequate vitamin A status ensures optimal villus architecture and enterocyte function, which directly influences nutrient absorption capacity. The 2 kg/ton inclusion level may have provided sufficient retinol to support these epithelial maintenance functions, whereas the 1 kg/ton level, while adequate for basal requirements, may not have exceeded the threshold required for measurable improvements in protein digestibility.

Vitamin D_3_, the biologically active form 1,25-dihydroxyvitamin D_3_, increases the efficiency of intestinal calcium absorption by upregulating epithelial calcium transporters (Reddy & Jialal, 2022). This role in calcium metabolism has indirect but critical implications for protein digestion; calcium ions are essential cofactors for the stability and optimal activity of pancreatic proteolytic enzymes, such as trypsin and chymotrypsin, within the small intestine. Furthermore, calcium is required for the secretion of these enzymes from the pancreas. By enhancing calcium absorption, high-dose vitamin AD_3_E supplementation (2 kg/ton) may have improved the pancreatic enzyme activity and the luminal environment for proteolysis, thereby increasing the digestibility of crude protein. Silva et al. (2020) demonstrated that vitamin ADE supplementation in cattle successfully increased circulating 25-hydroxyvitamin D concentrations, confirming the bioavailability of supplemented vitamins. Although their study did not detect improvements in overall nutrient digestibility, the authors acknowledged that vitamin status was successfully elevated, which may create permissive conditions for digestive function under specific dietary contexts. Furthermore, the gastrointestinal epithelium undergoes rapid turnover and is particularly susceptible to oxidative stress, especially under intensive production conditions. At the higher supplementation level (2 kg/ton), α-tocopherol may have provided sufficient antioxidant protection to maintain enterocyte integrity and brush border enzyme activity (Reddy & Jialal, 2022), thereby facilitating more efficient protein digestion and amino acid absorption.

### 3.3 Blood parameters

The observed alterations in blood metabolite profiles following protected AD_3_E supplementation provide insight into the metabolic adaptations underlying improved growth performance in fattening lambs. The dose-dependent effects on glucose, total protein, and lipid fractions reflect the established physiological roles of vitamins A, D_3_, and E in intermediary metabolism, tissue accretion, and antioxidant defense. The significantly lower plasma glucose concentration (Table 4) in lambs receiving 1 kg/ton protected AD_3_E (Treatment 2) compared to both the control and the 2 kg/ton treatment (Treatment 3) warrants consideration of vitamin-mediated effects on glucose homeostasis. This finding aligns with observations by Nazari Darabkhani et al. (2024), who reported that although plasma glucose concentrations were not significantly affected by AD_3_E supplementation in their study, glucose levels were numerically lower in vitamin-supplemented groups and decreased over the experimental period from day 30 to day 60. The administration of AD_3_E supplementation in cows was associated with a reduced blood glucose concentration relative to non-supplemented groups (Toma et al., 2021). In contrast, blood glucose levels in Mediterranean buffaloes remained unaffected by vitamin supplementation (Verdurico, 2012).

**Table 4:** Effect of varying levels of protected vitamin AD_3_E supplementation on the blood biochemical parameters and liver enzymes of fattening lambs.

Blood parameter**	Treatments*			SEM	P-value
	1	2	3		
Glucose (mg/dL)	85.61^a^	56.94^b^	84.44^a^	9.60	0.02
Total protein (g/dL)	6.56^b^	6.88^b^	7.96^a^	1.67	0.03
Albumin	2.71	2.97	2.89	0.47	0.43
Creatinine	1.22	1.19	1.42	0.12	0.64
Triglycerides	89.04	97.84	109.34	14.85	0.18
Cholesterol (mg/dL)	67.22^a^	72.35^a^	52.22^b^	5.33	0.001
HDL	41.04	49.04	47.12	4.12	0.25
LDL	87.42^a^	67.22^b^	63.22^b^	6.34	0.01
ALP	166.45	189.76	159.76	14.73	0.58
ALT	22.90	21.00	27.13	2.17	0.40
AST	80.21	86.01	68.01	5.67	0.06

^a, b^Different letters in each row indicate significant differences at the (*P* < 0.05) level.

*Treatments 1, 2, and 3 represent, respectively: control without supplementation, diet containing 1 kg per ton of protected AD_3_E supplementation, and diet containing 2 kg per ton of protected AD_3_E supplementation.

**Normal ranges include glucose 50–80 mg/dL, total protein 6–7.5 g/dL, albumin 2.7–3.9 g/dL, creatinine 1–2 mg/dL, triglycerides 20–40 mg/dL, cholesterol 52–76 mg/dL, HDL 30–50 mg/dL, LDL 20–40 mg/dL, ALP 68–387 U/L, ALT 11–40 U/L, and AST 60–280 U/L (Jackson et al., 2002; Kanekoet al., 2008).

The biologically active metabolite of vitamin A, all-trans retinoic acid, has been shown to increase insulin sensitivity and suppress hepatic gluconeogenesis (Tobias et al., 2023). Dakshinamurti (2015) comprehensively reviewed that retinoic acid reverses insulin resistance through suppression of the phosphoenolpyruvate carboxykinase (PEPCK) gene while inducing glucokinase gene expression, effectively directing glucose toward utilization rather than production. This mechanism provides a plausible explanation for the reduced circulating glucose observed at the moderate supplementation level of AD_3_E (Table 4). Furthermore, Chen (2021) demonstrated that vitamin A status and retinoic acid receptor (RAR) activation significantly affect the expression of genes involved in hepatic glucose metabolism, with interactions between insulin and retinoic acid signaling systems playing a critical regulatory role. Vitamin D acts upon the pancreas to increase insulin secretion (Taneera et al., 2025). This vitamin d-mediated enhancement of insulin release would facilitate greater peripheral glucose uptake and utilization, potentially contributing to the reduced glucose concentrations observed (Table 4). The combination of vitamin A, which improves insulin sensitivity, and vitamin D, which enhances insulin secretion, creates a synergistic environment for efficient glucose disposal.

Recent evidence from Yu et al. (2024) demonstrated that vitamin A deficiency leads to impaired glucose-stimulated insulin secretion and loss of intestinal glucagon-like peptide-1 (GLP-1) expression. Restoration of vitamin A normalized these parameters through activation of the retinoic acid receptor β (RARβ) signaling pathway. This intestinal mechanism represents an additional route through which vitamin supplementation may influence glucose homeostasis. The absence of this hypoglycemic effect at the higher supplementation level (2 kg/ton) may reflect a threshold phenomenon or the complex interplay between vitamin status and other metabolic hormones. The concurrently elevated total protein at this level suggests that amino acids may have been directed toward gluconeogenesis to support enhanced protein synthetic demands.

The elevated total protein concentration in lambs receiving 2 kg/ton protected AD_3_E (Treatment 3) compared to both control and 1 kg/ton groups indicates improved nitrogen retention and enhanced protein anabolic capacity at the higher supplementation level, consistent with direct evidence from ovine vitamin E supplementation studies (Ismail Mahmud & Karadas, 2023). This finding is particularly significant given that albumin concentrations remained unaffected across treatments, suggesting the increase reflects changes in the globulin fraction or other protein components rather than hydration status. Total protein and globulin concentrations of lambs increased with AD_3_E vitamin injection, and this increase was greater at 4 ml per animal than in other groups (Habeeb et al., 2015). The elevated total protein may be due to an active role of vitamins in maintaining the osmotic pressure of cells, maintaining the process of protein and albumin synthesis, and increasing their effectiveness in the synthesis of cellular proteins (Habeeb et al., 2008). Ismail Mahmud and Karadas (2023) investigated vitamin E (alpha-tocopherol acetate) supplementation in Awassi lambs and reported that dietary vitamin E at 200 and 400 mg/lamb/day significantly increased total protein in serum while decreasing glucose concentration. This direct evidence from ovine research corroborates the present findings and confirms that vitamin E contributes to improved protein status in growing lambs. The antioxidant function of vitamin E contributes to protein status by preserving cellular structures involved in protein synthesis. Njeru et al. (1995) established that serum and liver tocopherol concentrations reliably reflect vitamin E intake in young cattle, confirming that supplemented vitamin E is bioavailable and incorporated into tissues where it can exert cytoprotective effects. This membrane-stabilizing function supports sustained protein synthetic capacity in hepatocytes and myocytes by reducing oxidative damage. The dose-dependent nature of this response—significant only at 2 kg/ton—suggests that while the lower supplementation level meets basal requirements, the higher level provides sufficient vitamin surplus to maximally stimulate protein anabolic pathways.

The reduced cholesterol and LDL concentrations across supplemented groups (Table 4) align with established hypocholesterolemic effects of vitamin E in lambs (Ismail Mahmud & Karadas, 2023) and vitamin A-mediated regulation of lipid metabolism (Chen, 2021). These metabolic adaptations provide a mechanistic explanation for improved growth performance and feed efficiency with protected AD_3_E supplementation. The hypocholesterolemic effect observed in the present study is consistent with previous findings in ovine models, where vitamin A intake was associated with reduced cholesterol concentrations (Li et al., 2015). Evidence from Śmiecińska et al. (2023) demonstrated that prolonged excessive intake of vitamin A is associated with reduced plasma cholesterol, a phenomenon attributed to the influence of elevated dietary vitamin A on hepatic cholesterol synthesis and catabolism. These modulations in blood metabolite profiles reflect the physiological necessity of vitamin AD_3_E supplementation for maintaining systemic homeostasis and supporting vital metabolic functions in production animals (Ferdouse et al., 2024). Retinoic acid regulates adipocyte differentiation and lipid homeostasis through nuclear receptor signaling, influencing both lipogenesis and lipid clearance (Chen, 2021). The reduced cholesterol and LDL at 2 kg/ton likely reflects enhanced vitamin A-mediated regulation of lipid metabolism, directing lipids away from circulation and toward productive purposes such as membrane synthesis in growing tissues.

The lipid-lowering effects may also operate through vitamin E's function as a lipophilic antioxidant that protects circulating lipoproteins from oxidative modification; by reducing lipid peroxidation, vitamin E enhances the clearance of LDL particles from circulation and prevents their accumulation (Ismail Mahmud & Karadas, 2023). They have also reported that vitamin E at 400 mg/lamb/day significantly decreased cholesterol concentration in Awassi lambs to 40.5 mg/dL compared to non-supplemented groups. This finding directly parallels the present results and confirms that vitamin E exerts cholesterol-lowering effects in growing lambs. Luan et al. (2023) also reported that supplementation of finishing bulls with 500 IU/day of vitamin E resulted in decreased serum cholesterol concentrations and improved antioxidant status.The authors attributed this effect to the established role of vitamin E in inhibiting lipid peroxidation within circulating lipoproteins, thereby modulating lipid metabolism and enhancing oxidative stability.

The observation that triglyceride concentrations were not affected by vitamin supplementation is consistent with the findings of Njeru et al. (1995), who reported no vitamin E treatment effects on blood lipid fractions (cholesterol and triglycerides) in yearling cattle. This suggests that vitamins' effects on lipid metabolism may preferentially influence cholesterol homeostasis rather than triglyceride metabolism.

The differential responses observed between supplementation levels provide insight into the dose-dependent nature of vitamin effects on metabolism. The lower glucose at 1 kg/ton, coupled with elevated total protein only at 2 kg/ton, suggests distinct vitamin requirements for different metabolic pathways. Glucose homeostasis may be more sensitive to modest increases in vitamin status, potentially mediated through rapid effects on insulin signaling and intestinal incretin hormones (Dakshinamurti, 2015; Yu et al., 2024). In contrast, protein anabolism—requiring integrated effects on gene expression, hormone sensitivity, and cellular integrity—may necessitate higher vitamin intakes to achieve measurable improvements (Chen, 2021).

In the present study, the activities of alkaline phosphatase (ALP), alanine aminotransferase (ALT), and aspartate aminotransferase (AST) remained unaffected by AD_3_E supplementation. In contrast, Ismail Mahmud and Karadas (2023) reported that vitamin E-supplemented lambs exhibited reduced AST and ALT activities, suggesting enhanced hepatic functional status conducive to more efficient lipid metabolism. Similarly, in a study on goat kids, dietary vitamin E (up to 1000 IU) led to a significant reduction in both AST and ALT activities compared to a control. This was interpreted as an indication of improved hepatic function and reduced oxidative stress (Zayed et al., 2021). These findings suggest that in situations of metabolic stress or suboptimal health, vitamin E can have a measurable protective effect on the liver, lowering these enzyme markers. The absence of an effect in our study could be because the lambs were in a state of good health and low metabolic stress, meaning their baseline AST and ALT levels were already within a normal, unstressed range. Vitamin E's role is primarily antioxidant and cytoprotective, not directly enzyme-inhibitory (Zayed et al., 2021).

The most direct link between AD_3_E components and alkaline phosphatase (ALP) is through vitamin D. Research has shown that disturbances in vitamin D metabolism, such as d-hypervitaminosis, are accompanied by high activity of ALP in the blood serum (Velykyĭ et al., 2010). This is because vitamin D regulates calcium and phosphate homeostasis, and changes in its status directly affect osteoblast activity, which produces the bone isoform of ALP. Vitamin E was shown to influence the activity of enzymes responsible for activating vitamin D in the liver (vitamin D_3_ 25-hydroxylase) and, under conditions of excess vitamin D (D-hypervitaminosis), helped to normalize the mineral metabolism, contents of calcium, phosphates, and activity of alkaline phosphatase isoform in the blood serum (Velykyĭ et al., 2010). This suggests a complex interaction where vitamin E supports metabolic regulation. The lack of change in ALP in our experiment may indicate that the supplemented vitamins were not causing any adverse effects like cholestasis or a major, unregulated shift in bone remodeling. The stable ALP levels suggest a well-regulated mineral and bone metabolism in the supplemented lambs.

**Fig. 1:**
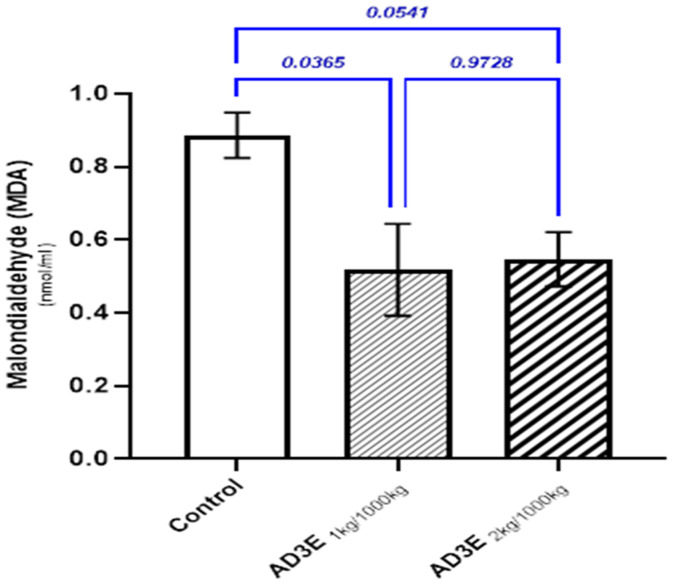
Effect of different levels of protected vitamin AD_3_E supplementation on blood malondialdehyde levels in fattening lambs.

**Fig. 2:**
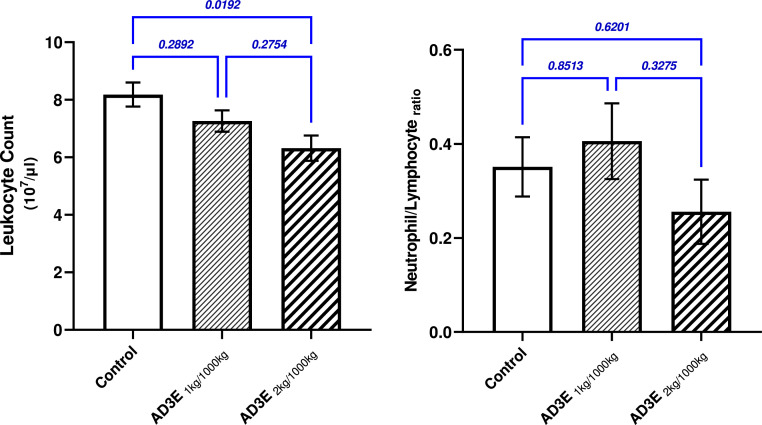
Effect of different levels of protected vitamin AD_3_E supplementation on leukocyte count and neutrophil to lymphocyte ratio in fattening lambs.

The decreased MDA index observed with protected AD_3_E supplementation in the present study provides clear evidence of reduced lipid peroxidation and improved antioxidant status in supplemented lambs (Fig. 1). This effect is primarily mediated by vitamin E's chain-breaking antioxidant activity within cellular membranes (Li et al., 2015; Simitzis et al., 2019), with potential synergistic contributions from vitamins A and D in maintaining cellular integrity and supporting overall antioxidant capacity (Strickland et al., 2021). The reduction in oxidative stress represents an important mechanism contributing to the improved growth performance and feed efficiency observed with AD_3_E supplementation (Fig. 2).

Table 5 demonstrates a significant increase in hematocrit (Hct) and hemoglobin (Hb) with protected AD_3_E supplementation, with higher values in Treatments 2 and 3 compared to the control (*P* < 0.05), suggesting enhanced erythropoiesis and oxygen transport capacity likely driven by vitamin D_3_ and E (Keywanloo et al., 2021). RBC count, MCV, MCHC, and platelet count showed no significant differences, indicating selective effects on red blood cell quality rather than quantity (Samarin et al., 2022). These outcomes imply that AD_3_E supplementation supports hematological health in fattening lambs without inducing polycythemia or thrombocytopenia.

**Table 5:** Effect of varying levels of protected vitamin AD_3_E supplementation on the hematology of fattening lambs.

Parameter^1^	Treatments			SEM	P-value
	1	2	3		
Hct (%)	23.21^b^	31.63^a^	29.74^a^	1.42	0.01
Hb (g/dL)	8.95^b^	10.12^ab^	11.20^a^	0.71	0.03
RBC (10^12^/L)	5.31	5.94	5.93	0.38	0.48
MCV (fl)	28.45	27.26	30.31	1.24	0.24
MCHC (g/dL)	32.57	33.53	33.59	1.05	0.76
Plt (10^9^/L)	565.11	646.62	527.63	47.26	0.13

^a, b^Different letters in each row indicate significant differences at the (*P* < 0.05) level.

Treatments 1, 2, and 3 represent, respectively: control without supplementation, diet containing 1 kg per ton of protected AD_3_E supplementation, and diet containing 2 kg per ton of protected AD_3_E supplementation.

^1^Normal ranges include Hct 27–45%, Hb 9–15 g/dL, RBC 9–15 × 10^6/μL, MCV 28–40 fL, MCHC 31–34 g/dL, and platelets 250–750 × 10^3/μL (Jackson et al., 2002; Kanekoet al., 2008).

Hemoglobin content varies with nutritional level (Kitchenham et al., 1975) because hemoglobin increases with increasing feed intake (El-Sabban et al., 1971). In the present study, lambs fed a diet supplemented with AD_3_E also had higher hemoglobin concentrations. It is reported that changes in protein intake are one of the factors affecting blood composition, because hemoglobin responds to changes in protein intake (Treacher, 1978). The increased Hct and Hb in supplemented groups align with a 2021 study on Holstein bulls, where parenteral vitamin D_3_ (22,000 IU/kg) significantly elevated hemoglobin and hematocrit in a dose-dependent manner, attributed to vitamin D's role in erythrocyte maturation (Keywanloo et al., 2021). Similarly, a 2022 study on growing lambs with organic trace minerals (including synergies with vitamins) reported higher erythrocyte counts and osmotic resistance, supporting improved hematology akin to AD_3_E effects here (Samarin et al., 2022). In contrast, a study on pigs found 25(OH)D_3_ enhanced immunity via increased immunoglobulins but did not detail hematological shifts, though it implied broader systemic benefits that could indirectly support blood parameters (Upadhaya et al., 2023). It was reported that vitamin A deficiency causes a decrease in the production of T and B lymphocytes, impairs phagocytosis, increases infections, and consequently reduces the positive function of the immune system (Klasing, 2007). The protected AD_3_E supplement included 300 mg selenium/kg, synergizing with vitamin E as an antioxidant to enhance immune function and reduce oxidative stress in fattening lambs (Giadinis et al., 2000). Selenium's inclusion likely contributed to improved protozoa populations and hematology via antioxidant synergy with vitamin E (Novelli et al., 2023). It is reported that selenium and vitamin E in sheep caused antibody production and greatly increased the level of resistance in livestock (Giadinis et al., 2000). In another report, selenium and vitamin E consumption increased the immune response of livestock cells (Turner et al., 1990). The reason for the ability of vitamin AD_3_E to stimulate the immune system is associated with an increase in immunoglobulin (Al-Asadi et al., 2020). Haga et al. (2021) demonstrated that vitamin E supplementation in dairy cows mitigates oxidative stress and enhances erythrocyte membrane stability, which may account for the observed improvements in hematological parameters independent of platelet counts. These comparisons indicate the results of our study are substantiated by ruminant studies, with vitamin D_3_ appearing key for hematocrit and hemoglobin enhancements.

### 3.4 Ruminal fermentation parameters

The effects of varying levels of protected vitamin AD_3_E supplementation on ruminal fermentation parameters in fattening lambs are presented in Table 6. The data indicate that supplementation exerted selective, rather than uniform, effects on the rumen environment. Rumen pH remained stable across all treatments, ranging from 6.43 to 6.67 (P = 0.38), suggesting that the additive did not compromise ruminal buffering capacity. In contrast, ammonia-nitrogen (NH_3—_N) concentrations increased significantly (P = 0.01) in a dose-dependent manner. This elevation likely reflects enhanced proteolysis or microbial activity. While total volatile fatty acid (TVFA) concentrations were numerically higher in treatment 3 compared to treatment 2 and the control (p > 0.05). Notably, the individual molar proportions of acetate, propionate, butyrate, and the resulting acetate to propionate ratio remained unaffected (P > 0.05). Consistent with the VFA data, estimated methane production also showed no significant differences between. These results suggest that protected vitamin AD_3_E supplementation primarily modulates nitrogen metabolism in the rumen, without broadly altering volatile fatty acid profiles or gas production.

**Table 6:** Effect of varying levels of protected vitamin AD_3_E supplementation on the rumen fermentation parameters.

Fermentation parameters	Treatments			SEM	P-value
	1	2	3		
pH	6.43	6.67	6.52	0.14	0.38
NH_3_-N (mg/L)	119.86^c^	138.54^b^	162.32^a^	2.97	0.01
TVFA (mmol/L)	93.43^b^	95.28^ab^	97.37^a^	0.01	0.572
C_2_ (mol%)	56.58	55.73	55.52	0.1	0.435
C_3_ (mol%)	16.77	17.91	19.2	0.01	0.367
C_4_ (mol%)	6.52	6.43	6.23	0.56	0.085
C_2_/C_3_	3.37	3.11	2.98	0.01	0.058
CH_4_ (Eq 1) (Mj/d)	5.48	5.47	5.45	0.04	0.855
CH_4_ (Eq 2) (Mj/ d)	3.85	3.72	3.62	0.07	0.281

^a, b^Different letters in each row indicate significant differences at the (*P* < 0.05) level.

Treatments 1, 2, and 3 represent, respectively: control without supplementation, diet containing 1 kg per ton of protected AD_3_E supplementation, and diet containing 2 kg per ton of protected AD_3_E supplementation.

Eq (1): CH_4 (MJ/d)_ = 3.96(±1.18) + 0.561(±0.130) × DMI _(kg/d)_.

Eq (2):CH_4 (MJ/d)_ = 2.70(±1.38) + 1.16(±0.271) × DMI _(kg/d)_ – 15.8(±6.86) × EE _(kg/d)_.

The differential effects of vitamin AD_3_E supplementation on ruminal parameters observed in the current study—increased ammonia-nitrogen without concomitant changes in pH or volatile fatty acid profiles—reveal important insights into the selective modulation of rumen fermentation by AD_3_E. The elevation in ruminal NH_3—_N concentration with AD_3_E supplementation likely reflects enhanced proteolytic activity within the rumen microbial ecosystem. This finding aligns with the established understanding that ruminal ammonia is primarily generated through microbial protein degradation, involving ammonia-producing bacteria and protozoal activity (Song et al., 2023a). The vitamin complex may stimulate bacterial deamination or urease activity, as suggested by Al-Asadi et al. (2020), though the specific mechanisms warrant further investigation. Song et al. (2023b) demonstrated that vitamin A administration in lambs enhances crude protein content in muscle tissue and improves antioxidant capacity. While their study focused on intramuscular injection in newborn lambs rather than dietary supplementation in fattening animals, the observed improvements in protein metabolism suggest that vitamin A may exert systemic effects on nitrogen utilization. This supports the interpretation that the increased NH_3—_N observed in the present study could reflect enhanced protein degradation or altered nitrogen partitioning.

The absence of significant changes in ruminal pH and the stability of major volatile fatty acid proportions, despite increased NH_3—_N, indicates that AD_3_E supplementation modulates nitrogen metabolism without broadly disrupting carbohydrate fermentation. This pattern is consistent with reports that vitamin E influences specific metabolic pathways—such as conjugated linoleic acid synthesis—without broadly modifying VFA profiles in sheep (Nazari Darabkhani et al., 2024; Guerra-Rivas et al., 2016). Carvalho et al. (2024) similarly reported that 25-hydroxyvitamin D_3_ supplementation in beef cattle improved ruminal microbial efficiency without affecting pH or VFA proportions, suggesting that vitamin D may enhance the metabolic activity of rumen microbes without altering fermentation end-product profiles. This aligns with the current findings and supports the hypothesis that vitamins AD_3_E can stabilize ruminal fermentation while selectively enhancing nitrogen metabolism.

The selective increase in NH_3—_N without major VFA alterations has important implications for understanding how vitamin supplementation affects ruminal efficiency. Ammonia concentration reflects the balance between protein degradation and microbial protein synthesis. The observed increase could indicate either enhanced proteolysis or reduced ammonia utilization by microbes. The latter seems less likely given that VFA production—an indicator of overall fermentative activity—was maintained or increased. The elevation in total VFAs observed in the current study aligns with previous research demonstrating that vitamin supplementation enhances ruminal fermentation. Vitamins B, C, and D have been reported to increase dry matter degradability and fermentation efficiency (Witariadi et al., 2022; Astawa et al., 2011). Specifically, vitamin E has been shown to linearly increase total VFAs, acetate, and the acetate: propionate ratio in vitro (Hou et al., 2013; Wei et al., 2015). These increments in VFA production are often associated with improved organic matter digestibility and gas production (Getachew et al., 2004), providing a plausible mechanism for the current results.

The mechanisms by which fat-soluble vitamins influence ruminal fermentation remain incompletely characterized. Vitamin A and its metabolite retinoic acid possess several biological functions beyond their classical roles, including modulation of gene expression and cellular differentiation (Song et al., 2023a, 2023b). While these effects are typically studied in animal tissues rather than rumen microbes, it is plausible that vitamins directly or indirectly influence microbial community composition or metabolic activity.

### 3.5 Protozoa population

Table 7 shows the effect of different levels of protected vitamin AD_3_E supplementation on the rumen protozoa population of fattening lambs. The differential response of rumen protozoa populations to varying levels of protected vitamin AD_3_E supplementation, particularly the peak in total counts and *Entodinium* abundance at the moderate supplementation level (Treatment 2), reveals a nuanced interaction between fat-soluble vitamins and the rumen microfauna. The dominance of *Entodinium* across treatments is consistent with its established role as the most prevalent ciliate genus in the rumen, often constituting over 90% of the total protozoal population (Voia et al., 2011). The observation that protozoa populations were highest at the moderate supplementation level but declined at the highest level suggests a non-linear, dose-dependent response. This pattern may explain discrepancies in the literature regarding vitamin effects on protozoa. For instance, a study by Nazari Darabkhani et al. (2024) investigating different methods of AD_3_E supplementation in fattening lambs reported that protozoa counts were reduced by vitamin supplementation overall. However, the present study demonstrates that this effect may be contingent upon dosage, with moderate levels potentially creating a more favorable ruminal environment for protozoal proliferation.

**Table 7:** Effect of varying levels of protected vitamin AD_3_E supplementation on the protozoa population (N × 10^5^) in the rumen of fattening lambs.

Protozoa	Treatments			SEM	SEM
	1	2	3	2	1
Total protozoa (×10⁵ cells/mL)	79.14^b^	96.73^a^	81.80^b^	0.82	0.01
*Entodinium* (×10⁵ cells/mL)	75.27^b^	92.32^a^	87.61^b^	0.87	0.002
(×10⁵ cells/mL) *Ophryoscolex*	0.56	0.68	0.51	0.04	0.16
*Diplodinium* (×10⁵ cells/mL)	0.88	0.95	0.75	0.08	0.37
*Isotricha* (×10⁵ cells/mL)	0.95	1.24	0.82	0.03	0.26
*Dasytricha* (×10⁵ cells/mL)	1.47	1.52	1.10	0.04	0.12

^a, b^Different letters in each row indicate significant differences at the (*P* < 0.05) level.

Treatments 1, 2, and 3 represent, respectively: control without supplementation, diet containing 1 kg per ton of protected AD_3_E supplementation, and diet containing 2 kg per ton of protected AD_3_E supplementation.

The selective stimulation of *Entodinium* is particularly noteworthy. As the dominant genus, *Entodinium* plays a crucial role in starch degradation and overall rumen function (Park et al., 2018). The antioxidant properties of vitamin E may contribute to this effect. Research has demonstrated that anaerobic rumen protozoa, including *Entodinium caudatum*, can be successfully cultivated under otherwise inhibitory aerobic conditions when antioxidants such as ascorbic acid and glutathione are included in the media (Park et al., 2018). Vitamin E (α-tocopherol), as a potent lipid-soluble antioxidant, may similarly protect protozoal membranes from oxidative damage within the rumen ecosystem, thereby promoting their survival and proliferation. This protective effect is consistent with findings by Chaudhary et al. (2014), who reported that vitamin E supplementation partially protected rumen ciliate populations from the toxic effects of monensin in goats.

Studies conducted using rumen batch cultures have shown that vitamin E has a positive effect on the rumen fermentation pattern and protozoal population (Naziroglu et al., 2002), although these effects appear to be highly dependent on the form and dose of vitamin E used, as well as the type of diet. Furthermore, supplementation with 50 IU per day of vitamin E in the form of α-tocopherol acetate, using an in vitro rumen simulation technique, demonstrated that due to its antioxidant effect, vitamin E increased the protozoal population in the fermentation environment (Belanche et al., 2016).

In the present study, the protozoal population also increased, but the important question is why this occurred under conditions where rumen-protected vitamin E was used. It seems that, given the vitamin used was coated with palm oil, it was not completely protected in the rumen. Therefore, while the positive antioxidant effect of vitamin E on protozoal growth and activity—and consequently on increased nutrient digestibility has been demonstrated, it is necessary to improve the level of vitamin protection in future studies.

The observed increase in protein digestibility and lamb weight gain alongside elevated protozoal populations aligns with the recognized contribution of protozoa to feed degradation. Ciliate protozoa contribute to fiber and starch breakdown both directly, through their own cellulolytic and amylolytic enzymes, and indirectly, through interactions with other rumen microbes (Park et al., 2018). The stimulation of *Entodinium*, in particular, may enhance starch digestion, contributing to the improved digestibility noted in this study. The relationship between protozoa and methane production is complex and not strictly causal. It is well established that protozoa host methanogenic archaea on their surfaces and within their cells, facilitating interspecies hydrogen transfer and methanogenesis (Tober et al., 2024). Some estimates suggest that 9–37% of methanogens are associated with protozoa (Morgavi et al., 2012). However, the absence of a significant increase in methane production alongside the elevated protozoal population in the present study is not entirely unexpected. Morgavi et al. (2012) demonstrated that the relationship between protozoa and methanogenesis is "not a simple cause–and–effect relationship," showing that defaunation does not consistently reduce methane production and can, in some cases, increase it depending on the adaptation period and associated microbial community shifts. The presence of protozoa may alter fermentation pathways and hydrogen partitioning in ways that do not necessarily lead to linear changes in methane output.

The findings of this study both align with and diverge from previous research. The stimulation of protozoa by AD_3_E supplementation contrasts with reports that mineral-vitamin supplements did not affect protozoal populations (Astawa et al., 2011) and with the overall reduction in protozoa counts reported by Nazari Darabkhani et al. (2024) with AD_3_E supplementation. However, the protective role of vitamin E against factors that suppress protozoa is supported by Chaudhary et al. (2014). Furthermore, the ability of antioxidants to support the growth of anaerobic protozoa under challenging conditions provides a mechanistic basis for the stimulatory effect observed at moderate supplementation levels (Park et al., 2018). The peak response at the intermediate dose suggests an optimal range exists, beyond which high concentrations of vitamins may exert inhibitory effects, possibly through direct antimicrobial activity or by altering the rumen microenvironment in ways unfavorable to certain protozoal species. The absence of significant changes in other genera (*Ophryoscolex, Diplodinium, Isotricha, Dasytricha*) suggests that *Entodinium* may be particularly responsive to the conditions created by AD_3_E supplementation, highlighting the genus-specific nature of this interaction.

### 3.6 Carcass characteristics

The evaluation of carcass characteristics in fattening lambs supplemented with protected vitamin AD_3_E revealed no significant treatment effects on any measured parameter (Table 8). Live weights at slaughter, carcass weights, non-carcass components (wool and skin), and biometric measurements (carcass length, width, thigh length, hand length) remained statistically unchanged across supplementation levels. These results indicate that the improvements in live performance observed in this study (Table 2) did not translate into detectable changes in carcass yield or conformational traits. The reason why the slaughter weight (Table 8) is not significant, unlike the final weight of the animals (Table 2), is that we selected the three largest animals from each group, and the pre-slaughter weight data differ from the final weight data in Table 2."

**Table 8:** Effect of varying levels of protected vitamin AD_3_E supplementation on the carcass characteristics of fattening lambs.

Carcass characteristics	Treatments				P-value
	1	2	3	SEM	
Live weight (kg)	58.73	58.00	58.46	3.51	0.33
Post-slaughter weight (kg)	56.60	55.35	53.20	3.48	0.31
Carcass weight (kg)	30.33	30.33	28.93	2/32	0.54
Weight of wool and skin (kg)	6.00	5.46	5.66	0.42	0.17
Biometric characteristics					
Carcass length (cm)	94.00	91.66	83.33	13.3	0.33
Carcass width (cm)	23.00	23.33	23.33	3.30	0.92
Thigh length (cm)	44.00	44.66	45.00	2.58	0.28
Hand length (cm)	45.66	45.33	43.66	5.24	1.00

^a, b^Different letters in each row indicate significant differences at the (*P* < 0.05) level.

Treatments 1, 2, and 3 represent, respectively: control without supplementation, diet containing 1 kg per ton of protected AD_3_E supplementation, and diet containing 2 kg per ton of protected AD_3_E supplementation.

The absence of carcass effects aligns with a substantial body of literature examining vitamin supplementation in ruminants. Researchers evaluated water-soluble and fat-soluble vitamin blends (including ADE) in young Nellore bulls over a 170-day period and reported no significant effects on carcass traits, concluding that vitamin supplementation does not influence carcass characteristics in finishing cattle (de Andrade et al., 2023). This finding directly supports the observations in the present study and extends the generalizability of this phenomenon across ruminant species and production systems. Similarly, research specifically examining vitamin E supplementation in lambs has consistently reported minimal effects on carcass traits. Atay et al. (2009) investigated vitamin E supplementation in Karya male lambs and found no significant effects on carcass characteristics, despite some improvements in meat stability and a numerical enhancement in feed conversion efficiency. Studies in Awassi lambs have corroborated these findings, demonstrating that while vitamin E may positively influence meat quality attributes such as lipid oxidation, drip loss, and color stability during storage, it does not significantly alter carcass weights or dimensions (Macit et al., 2003). This dissociation between performance enhancement and carcass modification suggests that vitamins may exert their primary effects on metabolic efficiency and tissue quality rather than gross carcass morphology.

The present findings are also consistent with research on protected nutrient technologies. Behan et al. (2019), in a comprehensive review of rumen-protected fat supplementation in dairy animals, noted that while protected nutrients enhance energy intake and improve body condition, they do not consistently alter carcass traits. This parallel between protected fats and protected vitamins suggests that rumen-protected delivery systems may prioritize metabolic efficiency and productive performance without necessarily redirecting nutrients toward carcass deposition. The lack of carcass response in the present study contrasts with emerging evidence that vitamin D metabolites can influence carcass characteristics in cattle. Carvalho et al. (2024) demonstrated that supplementation with 25-hydroxyvitamin D_3_ in finishing Nellore bulls increased carcass dressing percentage (55.2%vs. 53.8%) and tended to increase Longissimus muscle area, without affecting marbling or yield grade. This effect was attributed to upregulation of genes associated with skeletal muscle growth, including insulin-like growth factor-1 (IGF1), IGF2, and mammalian target of rapamycin (mTOR; Martins et al., 2025). The discrepancy between these findings and the present study may reflect species differences (cattle vs. sheep), the specific vitamin D metabolite used (25-hydroxyvitamin D_3_ vs. standard vitamin D_3_), or dosage considerations.

Mudado et al. (2024) further reported that 25-hydroxyvitamin D_3_ supplementation in grazing Nellore young bulls resulted in animals being 8.0 kg heavier than unsupplemented controls, with greater increases in rib width, indicating enhanced carcass growth. These findings suggest that the form and level of vitamin supplementation, as well as the physiological stage of the animal, may be critical determinants of carcass response. In small ruminants, limited evidence suggests potential for carcass improvements under specific conditions. Kannan et al. (2024) reported that combined vitamin supplementation in goats improved carcass traits, including muscle mass increases of 8–10%, indicating that higher supplementation levels or specific combinations may influence carcass outcomes in small ruminants. The absence of such effects in the present study reinforces the concept of an optimal dosing window, where moderate supplementation may enhance live performance while higher levels are required to translate growth gains into measurable carcass benefits.

### 3.7 Intestinal histomorphometry

An evaluation of small intestinal histomorphology (Table 9) revealed no significant treatment effects on papilla length, papilla width, or crypt depth across the duodenum, jejunum, and ileum (P > 0.05). These findings indicate that protected vitamin AD_3_E supplementation, at the levels tested, does not markedly alter the macroscopic structure of the small intestine in fattening lambs. The absence of morphological change may suggest that basal nutritional adequacy was sufficient for maintaining intestinal architecture, or that AD_3_E exerted effects on functional parameters—such as motility, nutrient absorption, or microbiome interactions—rather than on gross structural features. Research has established that vitamins A, D_3_, and E can modulate intestinal motility and influence gut microbial populations, which are integral to maintaining gut health and function (Malaguarnera, 2020). The absence of significant morphological alterations in the small intestine following protected vitamin AD_3_E supplementation aligns with a growing body of evidence suggesting that the primary effects of fat-soluble vitamins on the gastrointestinal tract may be functional and immunomodulatory rather than structural, particularly in healthy, adequately nourished animals.

**Table 9:** Effect of varying levels of protected vitamin AD_3_E supplementation on the intestinal morphology of fattening lambs.

Parameter	Treatments			SEM	*P-value*
	1	2	3		
Duodenum					
Papilla length (μm)	588.2	629.53	617.26	11.1	0.19
Papial width (μm)	120.93	118.53	117.6	1.35	0.40
Cript depth (μm)	249	246.93	249.2	3.21	0.82
Jejunum					
Papilla length (μm)	455.6	459.46	457.8	1.56	0.40
Papial width (μm)	114.33	115.2	118	1.38	0.36
Cript depth (μm)	168.4	171.73	167.26	1.69	0.37
Ileum					
Papilla length (μm)	623.26	634.4	628.26	5.37	0.49
Papial width (μm)	130.6	127.4	125	1.32	0.12
Cript depth (μm)	259.66	260.53	254.06	3.12	0.48

^a, b^Different letters in each row indicate significant differences at the (*P* < 0.05) level.

Treatments 1, 2, and 3 represent, respectively: control without supplementation, diet containing 1 kg per ton of protected AD_3_E supplementation, and diet containing 2 kg per ton of protected AD_3_E supplementation.

The present findings are consistent with research demonstrating that vitamin D plays a pivotal role in gut homeostasis and microbiota regulation without necessarily altering intestinal architecture. Malaguarnera (2020) highlighted that vitamin D pathways are critically involved in gut homeostasis and that the vitamin significantly affects gut microbiota composition and its dynamic activities. This immunomodulatory role may occur independently of changes in villus height or crypt depth, supporting the interpretation that the absence of morphological change in the present study does not preclude functional benefits. Furthermore, Wang et al. (2022b) comprehensively reviewed the relationship between gut microbiota and calcium balance, noting that vitamin D interacts with intestinal flora and related factors such as short-chain fatty acids (SCFAs) and immune factors to influence host physiology. These interactions can enhance nutrient absorption and barrier function without necessarily manifesting as measurable changes in tissue dimensions.

While the present study found no morphological changes with AD_3_E supplementation, evidence from other experimental models demonstrates that vitamin E can indeed influence intestinal structure under conditions of physiological challenge. Shirpoor et al. (2006) investigated the effect of vitamin E on diabetes-induced intestinal changes in rats and found that diabetic animals exhibited significantly increased villus height, crypt depth, and muscular layer thickness—changes that were completely normalized by vitamin E supplementation. The authors attributed this protective effect to enhanced plasma antioxidant capacity and reduced lipid peroxidation, suggesting that vitamin E exerts its most pronounced structural effects when the intestine is under oxidative stress.

This finding is particularly relevant to interpreting the present null results: in healthy, non-stressed lambs with adequate baseline nutrition, the antioxidant capacity of vitamin E may be sufficient to maintain homeostasis without inducing measurable structural adaptations. The protective role of vitamin E under challenged conditions is further supported by Vijayalakshmi et al. (2005), who demonstrated that vitamin restriction in rats significantly increased intestinal epithelial cell apoptosis and compromised villus architecture, while vitamin E supplementation reversed these changes. The authors concluded that "increased tissue oxidative stress seems to mediate the vitamin-restriction-induced apoptosis of the IECs in rats," and that vitamin E, through its antioxidant properties, was particularly effective in restoring intestinal integrity.

Future investigations should consider including measurements of intestinal permeability, mucosal immunity markers, microbial community composition, and nutrient transporter gene expression to fully characterize the functional consequences of AD_3_E supplementation. Additionally, exploring the effects of these vitamins under conditions of physiological stress or suboptimal nutrition may reveal structural benefits that are not apparent in healthy animals.

## 4 Conclusions

The use of protected vitamins AD_3_E at a moderate level (1 kg per ton) significantly improves growth performance and protein digestibility in fattening lambs. The vitamin enhances growth performance by an additional 2.5 kg through increasing nitrogen metabolism in the rumen, elevating ammonia-nitrogen concentration, and selectively stimulating beneficial protozoal populations, particularly *Entodinium*, without disrupting ruminal pH, volatile fatty acid profiles, or methane production. Vitamin AD_3_E also improves hematological parameters, indicating reduced oxidative stress and increased red blood cell stability in lambs receiving the vitamin. However, these metabolic and performance improvements do not lead to changes in carcass characteristics or intestinal morphology. The findings of this research indicate that adding AD3E at a rate of 1–2 kg per ton enhances rumen function and systemic health of fattening lambs, ultimately increasing lamb growth by 2.5 kg, without inducing structural changes in carcass or intestinal tissues.

## Ethical statement

These experiments were conducted in accordance with established animal welfare guidelines, and all experimental protocols adhered to and were approved by the Ethics Committee of Razi University, Kermanshah, Iran (ethics approval number: IR.RAZI.REC.1402.052). After conducting the experiments and submitting a full report with details about the experimental methods to the relevant committee, the committee confirmed the certificate number IR.RAZI.REC.1402.063 that the study complies with all regulations, in your manuscript.

## Funding sources

This research did not receive any specific grant from funding agencies in the public, commercial, or not-for-profit sectors.

## CRediT authorship contribution statement

**Danyal Zarrin Kelk:** Visualization, Formal analysis, Data curation. **Mohammad Ebrahim Nooriyan Soroor:** Supervision, Project administration, Funding acquisition. **Fardin Hozhabri:** Validation, Supervision, Project administration, Formal analysis, Conceptualization. **Hadi Cheraghi:** Writing – original draft, Investigation. **Khoda Bakhsh Rashidi:** Visualization, Methodology, Conceptualization.

## Declaration of competing interest

The authors declare that they have no known competing financial interests or personal relationships that could have appeared to influence the work reported in this paper.

## Data Availability

The data that support the findings of this study are available from the corresponding author upon reasonable request.
